# Are online meatball restaurants in Indonesia committed to their declared Halal label?

**DOI:** 10.14202/vetworld.2024.778-784

**Published:** 2024-04-10

**Authors:** Retty Ikawati, Yuny Erwanto, Boyke R. Purnomo

**Affiliations:** 1 Doctoral Program in Islamic Economy and Halal Industry, Universitas Gadjah Mada Graduate School, Yogyakarta, Indonesia; 2 Department of Food Service Industry, Faculty of Economics and Business, Universitas Ahmad Dahlan, Yogyakarta, Indonesia; 3 Department of Animal Products Technology, Faculty of Animal Science, Universitas Gadjah Mada, Yogyakarta, Indonesia; 4 Department of Halal Science, Institute of Halal Industry and System, Universitas Gadjah Mada, Yogyakarta 55281, Indonesia; 5 Department of Management, Faculty of Economics and Business, Universitas Gadjah Mada, Yogyakarta, Indonesia

**Keywords:** halal authentication, halal supply chain, online food delivery, traceability

## Abstract

**Background and Aim:**

Halal restaurants participating in online food delivery services do not require halal certification. The Halal status of products through the Halal logo provides the consumer with information on the basis of which he decides to buy. Online transactions involve potential risks related to online processes, payment methods, and product quality. The aim of this study was to determine whether a declared Halal label is in accordance with the business processes implemented.

**Materials and Methods:**

Halal authentication of Gofood’s meatball partner products in Yogyakarta and Solo Raya determined the incompatibility of meatball ingredients. Sixty meatball samples were collected from Yogyakarta and 30 samples from Solo Raya. Halal certification test was carried out using the thermal cycle polymerase chain reaction method at Universitas Gadjah Mada Animal Husbandry Laboratory and the results were used to identify pork contamination in meatballs. The addition of pork or pork meatballs was used as a control.

**Results:**

Eight meatball restaurants in the Solo Raya and Yogyakarta areas were found to be contaminated with pig DNA. The results of the tracing materials and processes, i.e., the grinding stage, are critical because all samples were supposed to be made from beef. It is known from interviews that contamination with pig DNA at the milling stage was accidental.

**Conclusion:**

Restaurants that sell meatballs are committed to adhering to product labels that are 91.1% safe from pork contamination. The Halal and original beef labels were in accordance with their statements. This study highlights the concept of Halal authentication with traceability to overcome pork contamination in meat products.

## Introduction

During the pandemic, social restrictions changed the way restaurants served customers. Several changes, such as takeaway and delivery systems, disposable utensils [1], digital payments [2], as well as concerns about government policy regarding business hours [3] have been made. Manufacturers use digital marketing strategies, including social media and online food delivery (OFD) platforms, to connect with their target audiences [4]. Consumers choose online services based on user-friendliness, advantageous features, time-saving, and trust factors [5]. However, their views on safety risks and healthy and nutritious foods also affect their choices. The importance of healthy, nourishing, and Halal foods [3, 6] is growing, but there are concerns about potential price increases. Therefore, consumers with a high perception of food safety risks are likely to pay a premium. Similarly, consumers who are cognizant of a product’s halal status consider the presence of a halal label a sign of hygienic, safe, and healthy food [7]. Halal-certified food items indicate that Tayyiban is implemented in business operations [8]. One study has shown that Halal labels [9, 10] and Halal certification affect product purchases. The trustworthiness of Halal assurance systems (HAS) lies in their quality attributes, which consumers cannot verify directly before or after consumption [11]. Halal auditors, together with representatives of the Islamic Scholar and witnesses who gather relevant facts and data, are responsible for investigating a company’s Halal products [12].

Proof of food fraud can be obtained from Halal authentication, which detects non-Halal ingredients in the product. Halal authentication is a key analysis process for verifying and validating product information on labels according to the origin of the food and production processes. Potential halal assurances often arise from food quality risks without economic motives and food fraud with economic motives [13], which are closely related to food safety factors [14]. Food fraud is the deliberate or unintentional substitution, addition, alteration, or provision of incorrect information regarding products, food items, or packaging to maximize profits [15]. Halal food fraud involves irresponsible producers using non-Halal ingredients in processing, providing counterfeit Halal logos, physically contaminating Halal food, and using logistics services that do not comply with Sharia law [16]. Food fraud can adversely affect quality and food safety and damage business reputation and consumer confidence [17].

For producers of meat and processed products, the application for Halal certification is a formal business strategy. However, in reality, the OFD service application does not require Halal certification for producers who cooperate with them. The rules in the OFD system are still weak, and the government’s lack of enforcement of regulations in the labeling of food products in the online market has led to an increase in the number of food fraud cases [18]. When using online services, consumers encounter distance limitations and are unable to directly assess the freshness, cleanliness, hygiene, and sanitation of the restaurant [19]. The possibility of food adulteration in processed meat products is a concern due to halal slaughter requirements and the demand to store them separately from unclean meat and haram ingredients, namely pork and rat meat [20], with traceability principles in place [21, 22].

To improve the integrity and efficiency of the Halal supply chain, it is essential to implement Halal traceability. An effective traceability system can reduce wasted and withdrawn products by 50%–90% [23]. As regards the implementation of Halal, producers must comply with the eleven necessary criteria. Reporting critical activities is an essential part of this process. Critical halal points refer to activities vulnerable to illegal contamination throughout the supply chain, from breeders, distributors, slaughterhouses, and retailers to consumers [24]. All processed meat that is slaughtered has the potential for critical activity [25]. In addition to slaughtering, the milling process [26] and the use of additional ingredients [27] are another critical activity for meatball preparation. The materials and activities involved in critical activities are aligned with the 11 criteria outlined in the (HAS 23000), which serves as a guide to ensure the halalness of production [28]. According to [29], the critical control points (CCP) for meatballs are grinding meat, grinding ingredients, boiling, draining, and cooling. Every material and process carried out in critical activities must be free from potentially harmful contamination; online searches and Halal label information can verify the Halal status of the materials used [30].

Halal/Haram meatballs have always been a sensitive topic in Indonesia, where the majority of the population is Muslim [31]. It concerns not only Halal status but also ethical violations [32]. Meatball restaurants have made significant efforts to persuade customers and maintain their trust. Some restaurants that do not have a Halal certificate have their products independently labeled Halal to ensure that they are safe for consumption.

Because there are no requirements for Halal ownership to guarantee certification for potential partners in OFD services, this study aimed to use a Halal authentication test to determine whether there was a potential for fraudulent food practices in meatball products produced by meatball restaurants in OFD applications. Is Halal’s logo certified, or is it a logo that is independently embedded in the business processes carried out? From this research, we hope that meatball restaurants will become more aware of the Halal concept and be motivated to apply for Halal certification as a guarantee of the Halalness of their products.

## Materials and Methods

### Ethical approval

This study did not include live animal or human sample. All meatball samples were purchased from the open market, so ethical approval was not necessary in this study.

### Study period and location

This study was conducted from April 2022 to August 2023, during the pandemic, when the social restriction policy and stay at home, purchasing via OFD carries a higher risk. The government has officially revoked Indonesia’s Covid-19 pandemic status and upgraded the country to endemic status on June 21, 2023. The government emphasized that the new normal does not mean relaxation of social distancing. The research conducted during and after the pandemic. This research is aimed at finding out compliance with meatball restaurants’ Halal commitments to food delivery services labeled as Halal was established through authentication tests on *Gofood* online shop meatball partner products in Yogyakarta and Solo Raya. The meatball samples were taken from meatball restaurants that were affiliated with *Gofood* online shop. To ensure population representativeness, 10% of the online meatball shop population [33] should provide 60 samples for Yogyakarta and 30 samples for Solo Raya. Purposive sampling, specifically meatball samples including OFD service members, was used in the qualitative study. The sampling process was considered representative of the population according to the criteria identified by the respondents [33].

### Polymerase chain reaction (PCR) analysis

**Figure-1:**
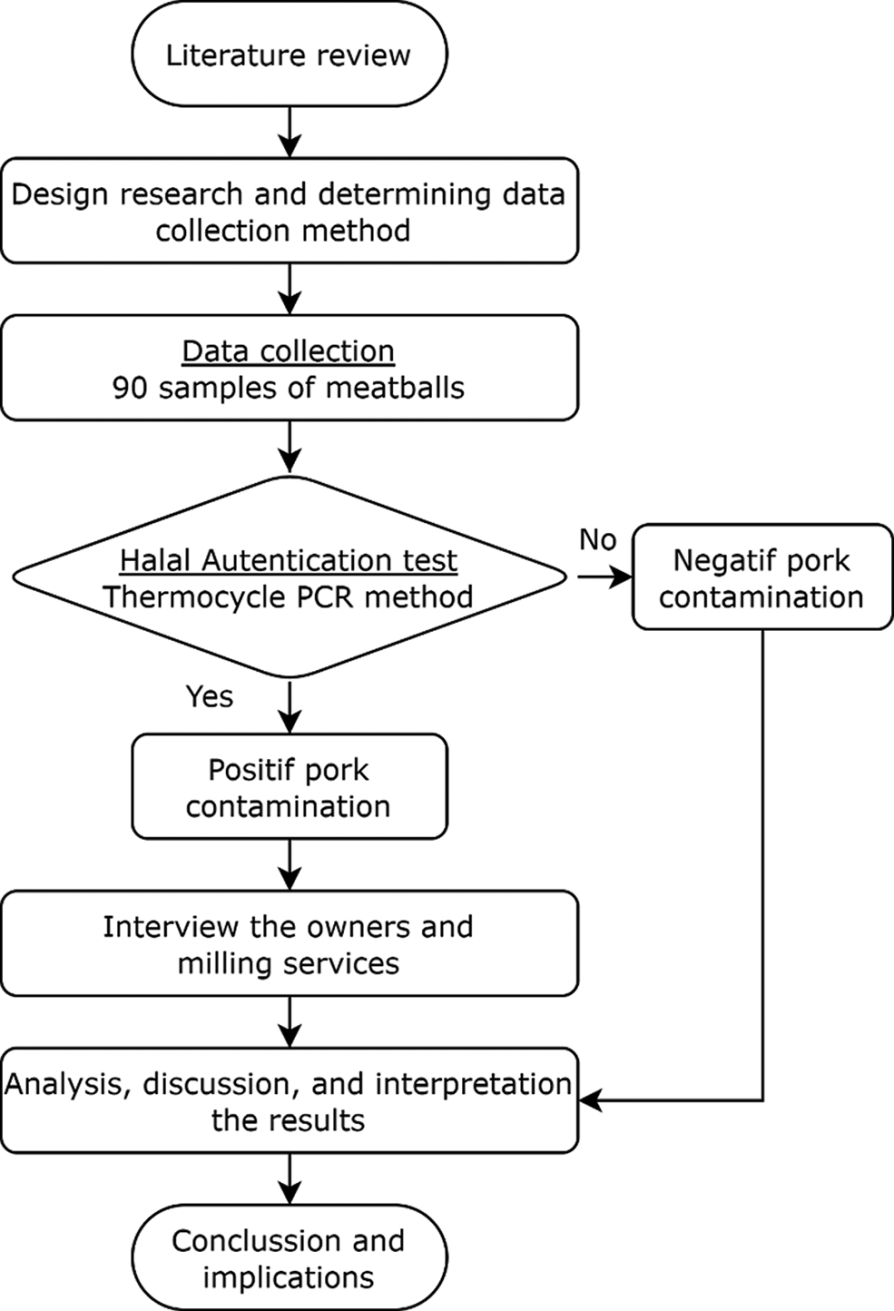
Research methodology flowchart.

Qualitative data included Halal authentication using the thermocycler PCR method, specifically in the analysis of laboratory findings regarding meatball products. The positive or negative status of pork contamination was determined from these results. The process of product authentication involved performing a pork contamination test on meatballs [34]. The results of the halal authentication test were analyzed through interviews with eight restaurants that were found to contain pork DNA. The positive status findings were then passed on to conduct interviews to trace ingredients and ground meat. To determine whether falsification, fraud, or any other irregularity had occurred, information on meat suppliers was used as evaluation material, together with meat supply chain data and laboratory test results. Figure-1 shows the flowchart of the study.

## Results

**Figure-2:**
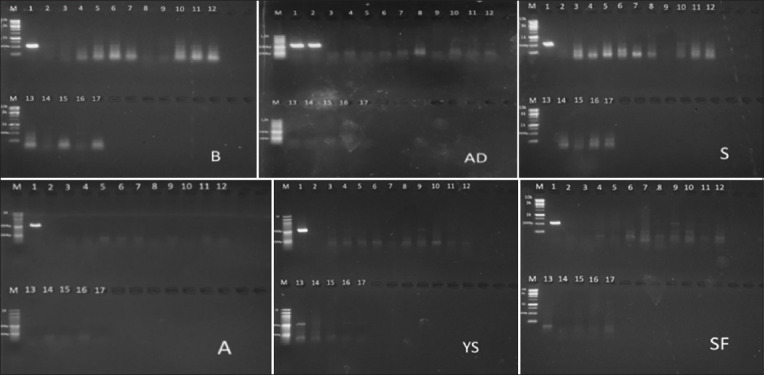
Pork contamination test results on 90 samples of meatballs using a cytochrome–b primer in the thermocycler polymerase chain reaction technique. The B samples from Boyolali area, AD samples from North-East Yogyakarta area , S samples from West Yogyakarta area, A samples from South Yogyakarta area, YS samples from Center Yogyakarta area and SF samples from Solo Sukoharjo area. Code 1 in each display as a positive control for pork. Code 2 as a negative control for beef. Codes 3–17 represent the tested meatball samples. The AD-1 and AD-2 bands run in parallel, except when the AD-1 code served as a positive control for pork and the AD-2 code served as a positive control for pork meatballs. The positive samples defined in samples B-06, B-10, B-11, B-12, AD-12, YS-09, YS-13, and SF-09.

Halal authentication of 90 meatball samples was performed at the Faculty of Animal Husbandry, Gadjah Mada University. Figure-2 describes the conditions of a meatball restaurant that contained pork DNA in the Halal authentication test. Code 1 represents a positive control for pork, code 2 serves as a negative control for beef, and codes 3–17 represent the meat samples tested. The AD-1 and AD-2 bands ran parallel, except when the AD-1 and AD-2 codes served as the positive and negative control, respectively. The interpretation of the other samples was positive if the resulting band aligned with the positive control code band 1 in each sample code. Samples B-06, B-10, B-11, B-12, AD-12, YS-09, YS-13, and SF-09 were defined as positive samples.

The results of the halal verification test showed that eight samples were positive for pork DNA, five from Solo Raya area, and three from Yogyakarta. Descriptive data from meatball sample owners tested for halal authentication included age, religion, educational background, micro-, small-, or medium-enterprise category, number of outlets, ownership of halal certificates for their meatballs, and labels listed independently by the outlets (Table-1). Halal certification status of samples indicated positive for pork DNA. Eight samples were then traced to raw materials and critical points according to the CCP criteria for meatball processing [29] (except for CCP 5, which is frying). Table-2 summarizes the compliance of each CCP with the standard. Milling (CCP 3) was the most vulnerable CCP stage because the status of the milling service used did not yet have a standard process for the Halal guarantee system. The samples were non-compliant for CCP 1 and CCP 3 as sago, salt, and a little cooking oil for boiling were not Halal certified. These results indicate that repairs and substitutions of materials are required to fulfill the Halal criteria for the CCP.

**Table-1:** Description of the owner of the meatball restaurant and the meatball Halal certification status of samples indicating positive for pork DNA.

No.	Sample code	Owner’s age	Religion	Education	Business category	Number of outlets	Halal certificate status	Label
1	B-06	18–29	Non-Muslim	Bachelor	Micro	3	Uncertified	Halal declared
2	B-10	50–64	Non-Muslim	Junior	Micro	1	Uncertified	Halal declared
3	B-11	50–64	Muslim	Senior	Micro	2	On Going	Real beef
4	B-12	50–64	Non-Muslim	Elementary	Micro	1	Uncertified	None
5	AD-12	30–39	Muslim	Senior	Small	1	Uncertified	None
6	YS-09	50–64	Muslim	Bachelor	Micro	1	On Going	Halal declared
7	YS-13	30–39	Muslim	Senior	Micro	3	Uncertified	Halal declared
8	SF-09	30–39	Muslim	Senior	Micro	2	Uncertified	Halal declared

**Table-2:** CCP tracking in samples indicated positive for pork DNA in each step on the flowchart.

No.	Sample code	Milling 1 (CCP)	Milling 3 (CCP1)					Boiling (CCP 3)	Draining and cooling (CCP 4)

			A	B	C	D	E		
1	B-06	✓	✓	✓	✓	✓	✓	✓	✓
2	B-10	✓	✓	✓	✓	✓	✓	✓	✓
3	B-11	✓	✓	✓	✓	✓	✓	✓	✓
4	B-12	✓	X	✓	X	✓	✓	X	✓
5	AD-12	✓	✓	✓	✓	✓	✓	✓	✓
6	YS-09	✓	✓	✓	✓	✓	✓	✓	✓
7	YS-13	✓	✓	✓	✓	✓	✓	✓	✓
8	SF-09	✓	✓	✓	X	✓	✓	✓	✓

CCP=Critical control point

## Discussion

For consumers, online transactions have a higher risk than offline transactions. The OFD service platform provider is not responsible for providing food but offers facilities such as a restaurant hygiene monitoring system, real-time order tracking system, and payment processing [35]. Consumers should be aware of online purchases to minimize potential risks, particularly with regard to product quality. The Halal concept challenges producers’ business performance. To gain consumer trust, producers must declare their products as Halal, especially processed meat products such as meatballs, which are a very sensitive issue in Halal meat. However, their meatballs are still uncertified. A product with a Halal label affects the consumer’s decision to purchase [9, 10]. However, they may become fraudulent if they do not comply with the actual conditions. Food fraud is defined as the act of intentionally or unintentionally making substitutions, additions, changes, or providing incorrect information regarding products, food ingredients, or packaging to maximize profits [15].

Of the eight meatball restaurants mentioned above, only two had a Halal certificate. Of these eight merchants, three were non-Muslim entrepreneurs. As mentioned in Table-1, meatball merchants have been grouped according to their status, micro, small, or medium-enterprise categories. Micro, small, and medium enterprises (MSME) owner’s religious background does not substantially affect its financial or non-financial performance [36]. However, the application of the Halal concept to small and medium-sized enterprises has a positive and significant impact on their business performance, especially for those with a religious background [37]. Adopting Halal is a business strategy for non-Muslim entrepreneurs to increase their revenue [38]. One merchant was a small enterprise, and seven others were micro-businesses. Small and micro-enterprises have a strong desire to obtain Halal certificates, but they are often hindered by administrative challenges and disorganized work systems [39]. It is difficult to address issues related to administration and recording as well as age and educational background. However, by aligning their restaurant with the OFD application, a recording process is initiated because a report is required. Non-Muslim entrepreneurs use Halal certification as a business strategy because they recognize that most customers are Muslims [38]. In spite of the fact that some restaurants do not have a Halal certificate, some restaurants claim that meatballs are Halal.

Halal products are a combination of tayyiban, which concerns the safety, nutritional value, and esthetic appeal of food products [40]. Figure-1 shows the results of the PCR test of the 90 meatball samples tested for Halal authentication. Eight samples contained pork DNA, indicating that 91.1% of meatball restaurants were free from contamination by haram ingredients, such as pork DNA. Three contaminated samples were obtained from Yogyakarta and five from Solo Raya. Solo Raya had 16.7% higher contamination rate than Yogyakarta (5%). On the basis of these results, an investigation was conducted for each meatball vendor. Traceability is one of the principles of the HAS and is implemented by tracking the ingredients used. Beef is one of the main ingredients used to prepare meatballs. The critical point requirements that were not met for halal materials, as shown in Table-2, were the use of additional auxiliary materials, such as sago, salt, and cooking oil, which did not have halal certificates, making their status doubtful. Two restaurants with signs of contamination applied for Halal certification and were aware of traceability requirements [41].

In addition to tracing critical points in processing raw materials, support, and assistance in the production of meatballs [27], the potential for critical Halal meatball activities can be identified through the supply chain of materials originating from the meat slaughtering process [42]. Slaughter, in accordance with Halal requirements, produces meat of the highest quality [43]. Although it has not yet been mentioned, the slaughter process is another critical point in the production of Halal meatballs. Although this variable is not a positive or a negative indicator in Halal authentication tests, the presence of pork DNA in the meatballs tested is important. However, in this study, only pork and beef were controlled and the potential addition of other meats could not be detected.

Great care is exercised in the selection of materials during the production process. MSME knowledge regarding halal certificates with religious factors drives the implementation of the Halal concept in Indonesia [37]. These businesses are aware that their products may be confronted with consumer questions concerning halal issues, including accusations of pork contamination [44]. However, the milling services used in the production cannot provide Halal certificates. The two merchants who used the same milling method did not have Halal certificates. While they claim that the milling service they use meets the requirements and accepts only milled chicken, beef, and fish and not pork, it does not provide evidence that the milling service does not perform the practice of milling illicit meat. Most meatball stalls choose milling services because they do not have their own milling machines. Milling services are more practical and save time and effort [45]. According to the information obtained from the milling services, only halal meat is accepted. However, there have been cases where pork has been included in milling machines in several cases since the owner is not in control of the process and employees are not able to control individual consumers who use the service. Some milling services have also tested positive for pork contamination [46].

The product label on the application or in the restaurant states that the meatballs are halal and made of genuine beef, but there is no information about the status of the meatballs. According to interviews with meatball restaurant owners, they have added other meats, including chicken, because of the high cost of beef without disclosing that the meatballs are not entirely made of beef. While the use of either beef or mixed ingredients such as chicken as raw materials is considered Halal, this practice is considered food fraud because it misleads consumers by substituting beef with cheaper alternatives [47]. This practice is motivated by the production cost factor [48]. Some respondents applying for a Halal certificate admitted that they did not take into account the milling section, which is a critical point that could violate consumer rights [26]. Milling service owners face problems in controlling new consumers and existing workers, which may lead to contamination beyond the required materials.

To overcome the challenges arising in this field, different approaches, such as strengthening traceability and halal supply chains, can be used. In the fight against food fraud, monitoring the supply chain before and after processing and within it is essential. Grinding is a crucial stage of meatball production and must be closely monitored. When controlling the milling process becomes problematic, consideration should be given to the factors that support the implementation of traceability and the Halal supply chain, as well as the economic and socio-cultural aspects that impact the success of the implementation [21]. The cluster concept can also be used to better organize the supply chain by grouping materials into haram and non-haram categories, making distinguishing and identifying materials easier. This strategy can be implemented through milling services and is supported by government policies and regulations. The government plays a critical role in maintaining the stability and quality of the Halal supply chain in a given area through regulations, financial incentives, taxation, infrastructure, and human resource training and education [49, 50]. For OFD, postproduction traceability is essential. For this reason, merchants who wish to participate in the industry must obtain Halal certification, which will soon be required by law. In addition, food suppliers must be trained in the halal supply chain to ensure the continuity of the supply chain until the product reaches the consumer [51].

## Conclusion

The restaurants in the current study were committed to the label attached to its product, which was 91.1% safe from pork contamination. The Halal and original beef labels were in accordance with the declarations. The remaining eight restaurants, five in Solo Raya, and three in Yogyakarta were contaminated with pork DNA. The results of the search for critical points showed that the grinding stage was the most critical because beef is the raw material used in all restaurants. Contamination of pork DNA by unintentional actions of producers is a critical stage in the milling process. As the main critical point of this research, it is necessary to conduct a more in-depth study regarding the application of Halal certification to milling services to provide guarantees for all business actors who use its services.

## Authors’ Contributions

YE, BRP, and RI: Conceived and designed the study. YE and BRP: Supervised this study. YE and RI: Sample analysis in the laboratory; BRP and RI: field research. All authors interpreted and analyzed the data drafted, read, reviewed, and approved the final manuscript.
